# Changes in Physical Activity in Relation to Body Composition, Fitness and Quality of Life after Primary Bariatric Surgery: a Two-Year Follow-Up Study

**DOI:** 10.1007/s11695-020-05009-x

**Published:** 2020-10-03

**Authors:** Malou A. H. Nuijten, Onno M. Tettero, Rens J. Wolf, Esmée A. Bakker, Thijs M. H. Eijsvogels, Valerie M. Monpellier, Eric J. Hazebroek, Ignace M. C. Janssen, Maria T. E. Hopman

**Affiliations:** 1 Radboud Institute for Health Sciences, Department of Physiology (392), Radboud University Medical Centre, P.O. Box 1901, 6500 HB Nijmegen, The Netherlands; 2 Nederlandse Obesitas Kliniek, Huis ter Heide, The Netherlands; 3 Research Institute for Sports and Exercise Sciences, Liverpool John Moores University, Liverpool, UK; 4 Department of Surgery, Rijnstate Hospital/Vitalys Clinics, Arnhem, The Netherlands

**Keywords:** Bariatric surgery, Physical activity, Body composition, Cardiorespiratory fitness, Quality of life

## Abstract

**Purpose:**

The success of bariatric surgery varies largely, which may relate to variance in adopting a physically active lifestyle. This study aimed to determine whether two-year changes in physical activity (PA) were associated with weight loss, fat-free mass, cardiorespiratory fitness and quality of life up to two years after bariatric surgery.

**Materials and Methods:**

In this retrospective study, 3879 post-bariatric patients were divided into three groups: 1) decreased PA (*n* = 388), 2) maintained PA (*n* = 2002) or 3) increased PA (*n* = 1498). Measurements regarding PA (Baecke questionnaire), body composition (bioelectrical impedance analysis), estimated cardiorespiratory fitness (Åstrand test) and health-related quality of life (RAND-36) were performed preoperatively and two years post-surgery.

**Results:**

Bariatric patients with increased PA had greater excess weight loss (76.3% vs. 73.2% vs. 72.9%, *P* < 0.001), greater increases in %fat-free mass (Δ14.0% vs. 13.0% vs. 12.8%; *P* < 0.001), larger improvements in VO_2_max (Δ11.8 vs. 10.2 vs. 8.0 ml/kg/min, P < 0.001), and larger increases in health related quality of life subscale scores (*P* < 0.05) compared to patients with maintained- and decreased PA.

**Conclusions:**

Bariatric patients who managed to induce improvements in habitual physical activity had better body composition, fitness and quality of life at 2 years post-surgery, compared to patients who maintained or even reduced their PA levels. These findings underscore the importance of perioperative-bariatric care programs to change lifestyle and achieve sustainable improvements in PA levels.

**Electronic supplementary material:**

The online version of this article (10.1007/s11695-020-05009-x) contains supplementary material, which is available to authorized users.

## Introduction

Bariatric surgery is considered the most efficient intervention for morbid obesity, showing promising results on weight control and reducing comorbidities (1, 2). However, 20–30% of the bariatric patients are not able to reach their targeted weight loss (1, 3), while 60% of the bariatric patients are not able to maintain at least 30% of their weight loss on the long term (4). This indicates a large diversity in effectiveness of bariatric surgery among patients, which persists after adjustment for different types of surgery (3).

The diversity in effectiveness of bariatric surgery may relate to variances in adopting a physically active lifestyle after surgery. Physical activity has a critical role in weight loss and long-term weight maintenance (5) and has a positive influence on body composition, cardiorespiratory fitness (CRF) and health-related quality of life (HRQoL) (6–8). Although previous studies with short-term exercise interventions after surgery show improved outcomes (9, 10), these well-controlled conditions do not reflect real-life setting including the effect of adopting an active lifestyle, which is necessary for long-term effectiveness (11).

We previously showed that sports activity was positively associated to CRF whereas leisure time activity was positively associated to weight loss (12). The present study builds further on these outcomes as we assessed the distinguished effects of either increasing or decreasing total PA on bariatric outcomes in a broader health spectrum. The aim of this study is to determine the relation between pre- to post-surgery changes in physical activity and weight loss, fat-free mass, CRF and HRQoL two years after surgery. We hypothesize that enhancing physical activity after bariatric surgery will contribute to the effectiveness of the surgical treatment on weight loss, body composition, CRF and HRQoL.

## Methods

### Study Population

Patient data with two years follow-up was prospectively collected in eight clinics of the Nederlandse Obesitas Kliniek (NOK), a Dutch Clinic that provides a comprehensive perioperative care program for patients with morbid obesity undergoing bariatric surgery. Patients were screened by an interdisciplinary team for eligibility for bariatric surgery based on the IFSO criteria (13). Inclusion criteria consisted of (i) a primary laparoscopic Roux-en-Y gastric bypass (RYGB) or sleeve gastrectomy (SG) which was performed between March 2012 and May 2015, (ii) participation in the preoperative care program with at least two years follow-up and (iii) complete data on physical activity. Patients who underwent a revisional bariatric operation or a gastric band operation were excluded.

### Perioperative Care Program

Before surgery, patients were enrolled in a 7-wk preoperative care program, provided by an interdisciplinary team consisting of a dietician, psychologist, physiotherapist and a physician, under the supervision of an internist and surgeon. The preoperative program consists of weekly group visits with an educational program on healthy lifestyle. After surgery, patients continue with a 15-month postoperative care program, with the aim to develop a healthy lifestyle without support of a bariatric care team. This program consists of group visits every three weeks, with each three consecutive 1-h sessions with a dietician, psychologist and physiotherapist. Furthermore, patients had regular individual medical check-ups with a physician and follow-up measurements (e.g. body composition, physical activity, CRF and HRQoL) up to 5 years post-surgery, to monitor the progress of the patient.

### Measurements

#### Physical Activity

Self-reported physical activity (PA) was assessed by the Baecke questionnaire. The Baecke questionnaire was modified to improve validity in this population with morbid obesity. Questions about sweating were excluded since obesity is related to excessive sweating. Therefore, sweating may decrease after surgery due to weight loss while in the questionnaire a decrease in sweating is seen as a decline in PA (14). Likewise, questions regarding the comparison of PA to peers were excluded, since obesity often occurs within specific layers of the community (15, 16). Therefore, our population might show similar behavior as peers within their environment, which the questionnaire interprets as sufficient PA, while the overall PA level within this environment might be poor.

The modified Baecke questionnaire contained 12 questions with a 5-point Likert scale on how much time was spent on several activities. Subsequently, three index scores (range 1–5 points) were calculated for work, sports and leisure time. These index scores were combined into a total Baecke score with a range from 3 to 15 points, in which a higher score represents a higher PA level.

#### Weight Loss

Weight loss was expressed in both percentage of total weight loss (%TWL) and percentage of excess weight loss (%EWL). %TWL reflects weight loss with respect to total preoperative weight, while %EWL reflects the weight loss with respect to preoperative excess weight (= preoperative weight minus appropriate weight based on a BMI of 25 kg/m^2^). Therefore, %TWL and %EWL can be calculated by the following formulas:

$$ {\displaystyle \begin{array}{c}\% TWL=\frac{weight\ loss\ (kg)}{preoperative\ weight\ (kg)}\ast 100\\ {}\% EWL=\frac{weight\ loss\ (kg)}{\left( preoperative\ weight\ (kg)-\left(25\ast {\left( height(m)\right)}^2\right)\right)}\ast 100\end{array}} $$

#### Body Composition

Height and waist circumference were measured using a non-elastic measuring tape. Weight, fat mass, fat-free mass (FFM, which mainly consists of muscle mass), fat percentage and FFM percentage (%FFM) were measured by a bioelectrical impedance analysis (TANITA® brand, model BC-420MA) (17).

#### Cardiorespiratory Fitness

Cardiorespiratory fitness (CRF) defined as VO_2_max (i.e. maximal oxygen uptake) was estimated by the Åstrand test, a submaximal exercise test on a cycle ergometer (18). In the first phase of the test, the resistance is increased every minute until a heart rate of ~120 bpm is reached. Then, the patient is instructed to continue cycling for six minutes at this resistance level, while the heart rate is continuously measured. After the test, the VO_2_max is estimated by combining patient characteristics (age and sex) with the achieved power output and the heart rate during the last 30s of the test. The Åstrand test could not be performed if subjects weighed ≥182 kg or used beta blockers or tricyclic antidepressants. Estimated VO_2_max was expressed in absolute VO_2_max (L/min) and VO_2_max relative to body weight (ml/kg/min) and FFM (ml/kg FFM/min). The absolute VO_2_max represents the maximal rate of oxygen consumption that the body can transport and metabolize within e.g. a minute during exercise, reflecting the aerobic capacity. However, this maximal oxygen consumption is also dependent on body type, therefore relative VO_2_max values takes body size into account to allow for comparison between individuals.

#### Health-Related Quality of Life

Health related quality of life (HRQoL) was determined by the RAND-36 questionnaire (19). The questionnaire contains 36 questions divided over eight subscales: physical functioning, physical role functioning, emotional role functioning, vitality, mental health, social functioning, bodily pain and general health. All subscales are expressed in a score between 0 and 100 points, in which a higher score reflects a higher HRQoL.

### Statistical Analysis

Statistical analyses were performed using SPSS 24 software (IBM SPSS Statistics for Windows, Version 24 IBM Corp., Armonk, NY, USA.). First, all continuous variables were visually inspected and tested for normality by the Shapiro-Wilk test. The population was divided into 3 groups based on their change in total Baecke score from pre-to post-surgery: a decreased PA group with >1 pt. decrease, a maintained PA group with a change between 1 and -1 pt., and an increased PA group with >1 pt. increase. Some practical examples of a > 1 point increase in total Baecke score are listed in supplemental Table 1. Pre-surgery characteristics were checked for differences between the groups by an ANOVA-test, Kruskal-Wallis test or Chi-Square test for continuous parametric-, continuous non-parametric- and categorical data, respectively. Then, pre- to post-surgery change in PA and health outcomes of the total population were determined by paired t-tests. Relation between change in PA and pre- to postoperative changes in outcomes (i.e. %EWL, %TWL, FFM (kg), %FFM, VO_2_max and HRQoL subscales) were assessed by an ANCOVA, adjusted for age, sex, type of surgery, preoperative BMI and preoperative physical activity. These covariates were included because of either a significant effect on the health outcome or based on rationale. Statistical significance was assumed at *P* < .05(two-sided).

## Results

**Fig. 1:**
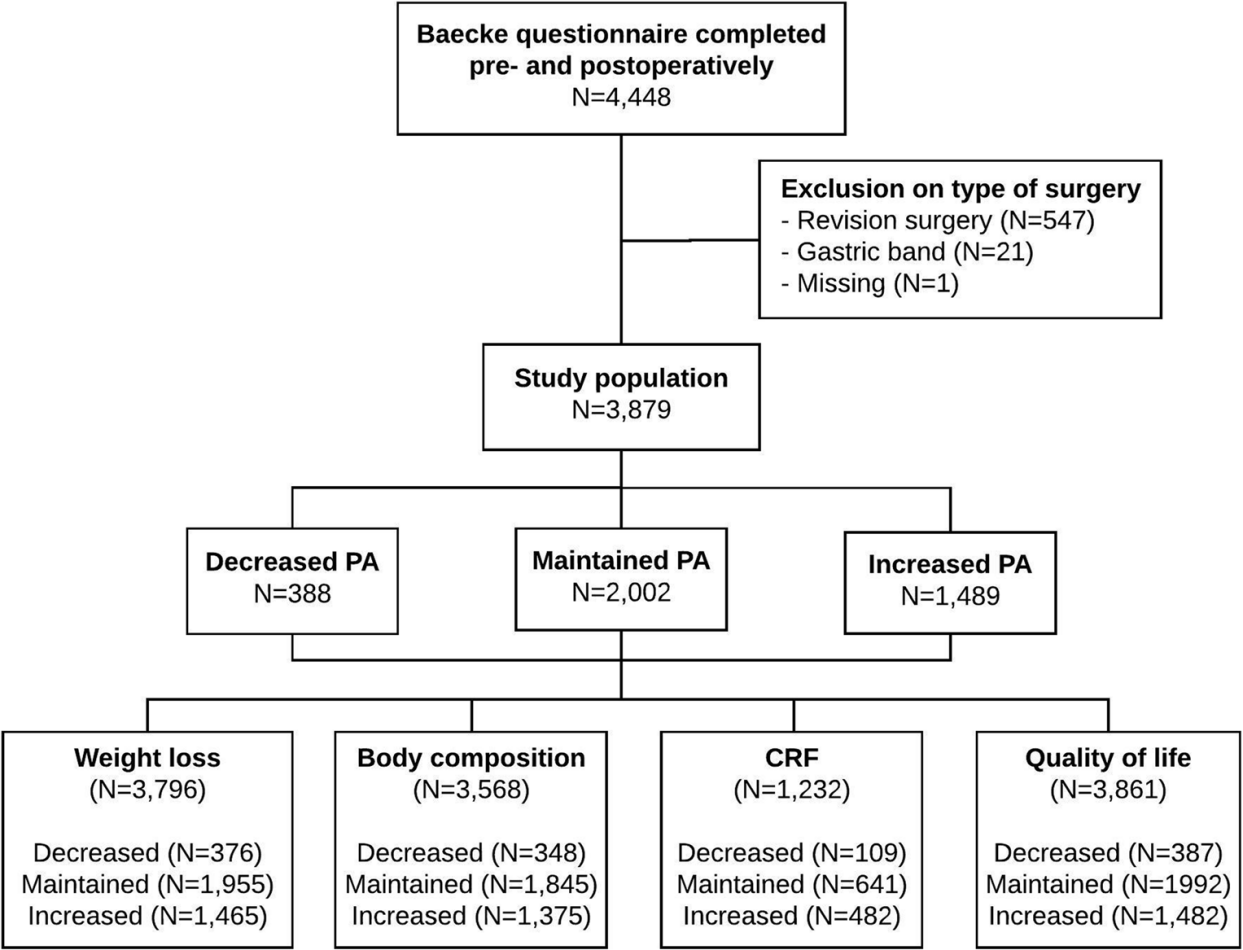
Enrollment of study population and compliance within outcome measures. CRF = cardiorespiratory fitness

A total of 4448 patients completed the Baecke questionnaire pre-surgery and two years post-surgery. Based on type of surgery, 569 patients (12.7%) were excluded, resulting in a study population of 3879 patients (Fig. 1). Based on the pre- to post-surgery change in PA, the population was divided into three groups: a decreased PA group (*n* = 388), a maintained PA group (*n* = 2002) and an increased PA group (*n* = 1498). With respect to missing values in outcomes, compliances were 96.8% for weight loss, 91.5% for body composition, 31.2% for CRF and 99.5% for HRQoL. Compliance rates were not significantly different between PA subgroups for each outcome measures (all *P* > 0.05).

### Pre-Surgery Characteristics

Pre-surgery characteristics of the total population and the three separate groups are displayed in Table 1. The increased PA group consisted of relatively more males, was significantly older and had a higher preoperative BMI compared to the maintained- and decreased PA group.

**Table 1:** Preoperative characteristics of total population and all groups based on change in physical activity

	**Total**	**Decreased PA** < -1 pt	**Maintained PA** − 1 to 1 pt	**Increased PA** > 1 pt	**P**
N	3879	388	2002	1498	
%Male	18.1	13.9	16.6	21.3	<0.001^a^
Age, y	43.9 ± 10.6	42.8 ± 11.4	43.7 ± 10.7	44.6 ± 10.1	0.005^a^
BMI, kg/m2	43.4 (40.4–47.9)	41.8 [39.2–46.0]	43.1 [40.3–47.2]	44.3 [40.1–49.4]	<0.001^a^
Type of surgery, n(%)					
RYGB	3552 (92)	361 (93)	1821 (91)	1370 (92)	0.297
SG	327 (8)	27 (7)	181 (9)	119 (8)	
Smoking, n(%)					
Yes	528 (13.6)	58 (14.9)	279 (13.9)	191 (12.8)	0.657
No	2128 (54.9)	221 (57.0)	1092 (54.5)	815 (54.7)	

Parametric values are means ± SDs and non-parametric values are median[IQR]. *P* values were derived by an ANOVA test for parametric variables, by a Kruskal-Wallis test for non-parametric variables and by chi-square test for categorical variables. RYGB = Roux-en-Y gastric bypass, SG = Sleeve gastrectomy

^a^significantly different between all groups

### Pre- to Post-Surgery Changes

For the total group two years after surgery, %TWL and %EWL were 31.4 ± 8.9% and 74.3 ± 22.9%, respectively (Table 2). Time-dependent changes in weight loss were displayed in supplemental Fig. 1. Absolute FFM decreased with 9.2 ± 5.9 kg (*P* < .001), whereas the %FFM increased with 13.4 ± 6.6% (*P* < .001). Absolute VO_2_max showed a mean increase of 0.10 ± 0.60 L/min whereas VO_2_max relative to FFM increased by 7.6 ± 10.1 ml/kg FMM/min (both P < .001). All RAND-36 subscale scores were significantly higher two years post-surgery compared to pre-surgery scores (all *P* < .001), with the greatest improvements in the vitality- and general health subscales (+34.4 ± 25.1 pt. and + 40.6 ± 36.4 pt., respectively).

**Table 2:** Pre- to two years post-surgery changes in health outcomes in the total population (*n* = 3879)

	**Pre-surgery**	**Post-surgery**	**Change (95%CI)**	**P**
Weight, kg	128.7 ± 21.3	88.2 ± 18.2	−40.5 (40.1–41.0)	<0.001
BMI, kg/m^2^	44.6 ± 6.0	30.6 ± 5.4	−14.1 (13.9–14.2)	<0.001
%TWL	NA	31.4 ± 8.9	NA	NA
%EWL	NA	74.3 ± 22.9	NA	NA
Fat-free mass, kg	61.6 [56.9–69.1]	52.5 [48.5–58.9]	−9.2 (9.0–9.4)	<0.001
%FFM	49.8 [47.4–52.5]	63.1 [58.2–69.4]	13.4 (13.1–13.6)	<0.001
**Cardiorespiratory fitness**				
VO_2_max, L/min	2.37 [1.97–2.85]	2.50 [2.09–2.96]	0.10 (0.07–0.14)	<0.001
VO_2_max, ml/kg/min	19.2 [15.7–23.1]	29.5 [24.1–35.2]	10.7 (10.3–11.0)	<0.001
VO_2_max, ml/kg FFM/min	39.3 [32.4–46.2]	46.2 [38.8–54.8]	7.6 (7.0–8.2)	<0.001
**HRQoL**				
Physical functioning	100 [67–100]	100 [100–100]	5.6 (4.1–7.0)	<0.001
Physical role functioning	75 [50–88]	87.5 [62.5–100]	12.4 (11.5–13.3)	<0.001
Emotional role functioning	48.8 ± 18.0	62.6 ± 20.6	13.8 (13.1–14.5)	<0.001
Vitality	51.7 ± 23.9	86.0 ± 21.1	34.4 (33.6–35.2)	<0.001
Mental health	72 [60–84]	80 [64–88]	5.0 (4.4–5.6)	<0.001
Social functioning	58 [45–78]	90 [57.5–100]	17.6 (16.7–18.5)	<0.001
Bodily pain	43.5 ± 18.1	70.0 ± 20.4	26.6 (25.8–27.3)	<0.001
General health	25 [25–50]	75 [50–100]	40.6 (39.4–41.7)	<0.001

Parametric values are means ± SDs and non-parametric values are median[IQR]. P values were derived by a paired samples t-test for parametric variables, and by Wilcoxon signed-rank test for non-parametric variables. TWL = total weight loss, EWL = excess weight loss, VO_2_max = maximal oxygen uptake, FFM = fat-free mass, HRQoL = health-related quality of life

**Fig. 2:**
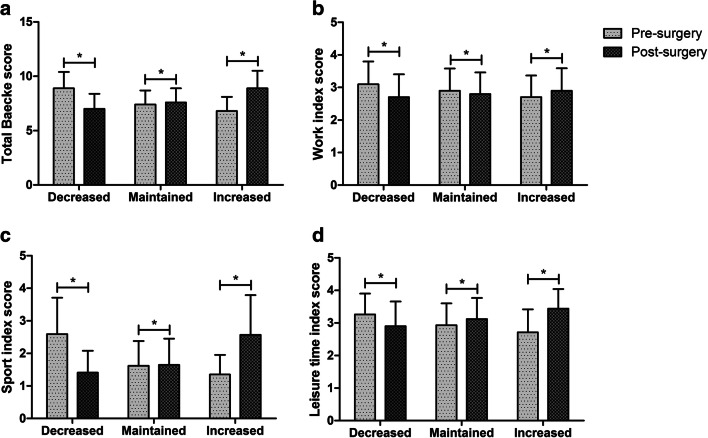
Modified Baecke scores pre- and two years post-surgery for the increased-, maintained and decreased PA subgroups. (A) Total Baecke scores (B) Work index scores (C) Sport index scores (D) Leisure time index scores. Bars reflect means with standard deviation. * *P* < 0.05

Regarding PA, the total group showed an increase of 0.7 ± 1.5 point on the total Baecke score from pre- to two years post-surgery (*P* < .001), with significant increases in all index scores (all P < .001). When considering the separate groups, the decrease group showed a decrease of 1.9 ± 0.8 points in total Baecke score, whereas increases of 0.2 ± 0.5 point and 2.2 ± 1.0 points were found for the maintained- and increased PA group, respectively (Fig. 2a). When expressing these PA changes in terms of walking activity, changes were comparable to increases of 38 ± 26 min/week for the increased PA group and 3 ± 19 min/week for the maintained PA group, and a decrease of −33 ± 24 min/week for the decreased PA group. The three groups showed similar patterns of changes in physical activity within the three index scores (i.e. work, sports and leisure time; see Fig. 2b, c and d).

### Physical Activity and Outcome Parameters

**Fig. 3:**
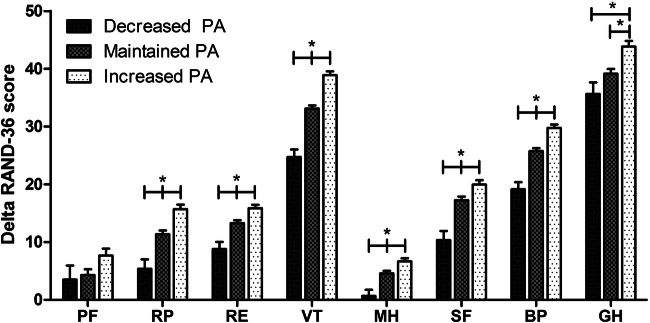
Change in health-related quality of life subscales (Delta Rand-36) from pre- to post-surgery adjusted for age, sex, type of surgery, pre-surgery BMI and pre-surgery physical activity. Bars reflect mean change with standard error of the mean. PF = physical functioning, RP = physical role functioning, RE = emotional role functioning, VT = vitality, MH = mental health, SF = social functioning, BP = bodily pain, GH = general health

The effects of change in PA after bariatric surgery on weight loss, body composition and estimated CRF are summarized in Table 3. Increasing PA was related to 1.4% more TWL, 3.1% more EWL, 0.96% more improvement in %FFM and 1.6 ml/kg/min more improvement in VO_2_max compared to the maintained PA group. These improvements were even greater compared to the decreased PA group (1.7% more TWL, 3.4% more EWL, 1.16% and 3.9 ml/kg/min more improvement in %FFM and VO_2_max relative to body weight). In contrast, the decreased PA group showed a significant attenuation of CRF outcomes (−0.19 L/min, −2.2 ml/kg/min and − 4.0 ml/kg FFM/min), compared to the maintained PA group. The absolute VO_2_max (L/min) in the decreased PA group was even lower compared to preoperative measures (pre: 2.65 ± 0.66 L/min vs. post: 2.53 ± 0.65 L/min). Furthermore, the increased PA group showed greater improvements in all subscales of the RAND-36, except for physical functioning (all *P* < .05), whereas the decreased PA group showed significantly less improvement in RAND-36 subscales compared to maintained PA group, except for physical functioning and general health (see Fig. 3). No effect of changes in PA were found on FFM (kg) (*P* = 0.324). All responses were similar between sexes (see supplemental Fig. 2).

**Table 3:** ANCOVA of differences in outcome parameters between physical activity groups

	**Decreased PA**		**Maintained PA**		**Increased PA**		**Group-effect** (*P* value)
	*N*	Mean (SE)	*N*	Mean (SE)	*N*	Mean (SE)	
**%TWL**	*376*	30.6 (0.5)	*1955*	30.9 (0.2)	*1465*	32.3 (0.2)	<0.001^a,b^
**%EWL**	*371*	72.9 (1.2)	*1929*	73.2 (0.5)	*1455*	76.3 (0.6)	<0.001^a,b^
**FFM** (kg)	*348*	−8.9 (0.3)	*1845*	−9.1 (0.1)	*1375*	−9.4 (0.2)	0.324
**%FFM** (%BW)	*358*	12.8 (0.4)	*1889*	13.0 (0.1)	*1412*	14.0 (0.2)	<0.001^a,b^
**VO**_**2**_**max** (L/min)	*109*	−0.11 (0.06)	*636*	0.09 (0.02)	*480*	0.17 (0.03)	<0.001^a,b,c^
**VO**_**2**_**max** (ml/kg/min)	*109*	8.0 (0.6)	*634*	10.2 (0.3)	*479*	11.8 (0.3)	<0.001^a,b,c^
**VO**_**2**_**max** (ml/kg FFM/min)	*107*	3.3 (1.0)	*628*	7.3 (0.4)	*468*	8.9 (0.5)	<0.001^a,b,c^

Data are presented as adjusted mean (SEM). Covariates included in this analysis were age, sex, type of surgery, preoperative BMI and preoperative total Baecke score. TWL = Total weight loss, EWL = Excess weight loss, FFM = Fat-free mass, BW=Body weight, VO_2_max = Maximal oxygen uptake

^a^Different between the increased- and maintained PA group; ^b^ Different between the increased- and decreased PA group; ^c^ Different between the maintained- and decreased PA group

## Discussion

We assessed the association between two-year changes in physical activity levels and health outcomes in post-bariatric surgery patients. In addition to our previous work (12), we revealed that only 38% of bariatric patients had substantially increased physical activity levels at 2 years post-surgery. These patients had greater weight loss and greater improvements in body composition, CRF and HRQoL compared to the patients who did not change or decreased their physical activity. Our findings underscore the importance of perioperative bariatric care programs to induce sustainable lifestyle improvements and improved habitual physical activity levels.

We found a positive relation between improvements in physical activity and weight loss following bariatric surgery. The magnitude of weight loss among the increased PA group (−43.0 ± 14.7 kg) was lower compared to findings reported in a previous study (−52.5 ± 15.4 kg) (20). However, this study defined increased physical activity by reaching physical activity guidelines and therefore probably also reflects greater increases in PA compared to our study. We defined change in activity as the difference between pre- and post-surgery physical activity rather than reaching specific cut-off values (e.g. reaching physical activity guidelines), since small increases in physical activity can yield large health benefits (21–23). Our findings confirm that small PA improvements are associated with better long-term outcomes, which could be highly motivating for post-bariatric patients to become more active. Furthermore, a meta-analysis on the effect of exercise interventions on post-bariatric weight loss also showed 1.94 kg additional weight loss in exercise groups compared to controls (24). However, these well-controlled conditions of supervised training programs can often not be reproduced in unsupervised real-life conditions, and do therefore not reflect adoption of a more active lifestyle. Nevertheless, two-year changes in PA are of the highest importance, since patterns of post-bariatric weight loss suggest that improvements up to 1–1.5 years post-surgery are ascribed to the impact of the procedure, whereas preservation of improvements is related to changes in lifestyle (25). This pattern is supported by our data, since peak weight loss occurred around 15 months, whereas weight loss ranges became substantially larger around 2 years (supplemental Fig. 1). The larger inter-individual variation is likely the result of substantial differences in lifestyle factors, such as PA. Therefore, further improvements (and possibly less weight regain) are expected when increased PA levels can be sustained. Taken together, our findings promote the value of post-bariatric care programs for achieving changes in lifestyle and improvements in PA levels.

Preservation of FFM is becoming a relevant topic in post-bariatric care, because of the essential role of FFM in several metabolic processes and health. In contrast to our hypothesis, no association between physical activity and absolute FFM (kg) was found. This might be explained by the strong association between FFM loss and weight loss, and the large variation in weight loss within our population (26, 27). Although excessive loss of FFM tissue might be harmful for underlying metabolic processes, eventually restoration of a health body composition (i.e. a higher %FFM) is probably more important than preservation of absolute FFM (kg) (28), because of its strong relation to several cardiovascular risk factors (29). Our results suggest that increasing physical activity is related to greater improvement in %FFM. Therefore, increasing physical activity could have a positive impact on cardiometabolic health, by achieving a relatively larger muscle mass.

Another valuable finding of this study is the strong association between physical activity and CRF. CRF is an important predictor of mortality risk (30) and it is known that people with morbid obesity suffer from a poor CRF (31). Although all PA groups showed improvements in relative VO_2_max (ml/kg/min) (i.e. relatively more ml of oxygen available per kilogram body weight), absolute VO_2_max (L/min) was exclusively improved by maintained- or increased PA. suggesting an increase in maximal oxygen uptake and a gain in aerobic capacity during exercise. The decreased PA group showed a reduction in absolute VO_2_max (L/min), suggesting that no real aerobic gain was obtained in this group. These findings highlight the essential role of physical activity in CRF, considering that weight loss on itself is not sufficient to improve physiological measures of CRF (32). A large meta-analysis showed that even a small increase of 3.5 ml/kg/min of oxygen consumption in CRF (corresponding to e.g. 1-km/h higher walking speed) is already associated with a 15% and 13% reduction in all-cause and cardiovascular mortality, respectively (33). Thus, the 3.9 ml/kg/min greater increase in CRF from our increased PA group reflects a substantial gain in health compared to the decreased PA group.

To the best of our knowledge, this is the first observational study that examined the relation between physical activity and HRQoL at more than 12 months post-bariatric surgery. Some previous studies assessed HRQoL and physical activity within the first year post-surgery (20, 34), however a longer follow-up is needed to assess the effects of adopting a more active lifestyle. Our large cohort of patients with 2 years follow-up demonstrated that an increase in physical activity is related to greater improvement in both physical- and mental health. Especially the positive association with the mental components of HRQoL is interesting, since bariatric surgery on itself does typically have a greater impact on physical aspects compared to mental aspects (35). Therefore, physical activity could potentially contribute to an improved mental well-being in these patients, next to the general physical benefits.

It is unclear why some patients are able to improve their habitual physical activity levels and others are not, despite the same post-bariatric care program. The present study showed substantial impact of patient characteristics on the ability to improve PA, but the increased PA group showed a higher prevalence of hypertension, sleep apnoea and back complaints. Furthermore, the increased PA group was significantly more inactive before surgery, considering that 43% walked or cycled less than 15 min per day and only 9% participated regularly in sports. Therefore it is easier to improve PA for these patients by engaging in sports activities or increasing walking time. Previous studies also suggest other barriers to exercise in post-bariatric populations, such as physical barriers and discomfort, self-presentational concerns, resources, time, environment, and intrinsic motivation (36–38). It is likely that these factors played a role in our study as well, therefore focusing on these aspects might improve post-bariatric care.

One limitation of our study is the relatively short follow-up of two years, since this period is considered the very end of the honeymoon period at which failure rates start to rise. Therefore, greater impact of life style factors such as PA would be expected with longer follow up. Nevertheless, the follow-up of 2 years is still interesting because our population passed the tipping point of weight loss (39) and patients entered the normalization phase in their personal environment. Further research into longer-term effect of bariatric surgery, physical activity and weight loss is warranted. Another limitation is the use of self-reported data for physical activity, because self-reported data can be influenced by social desirability and is less valid and reliable than objective measures (40). However, by including longitudinal data, our study obtains more insight into relations between physical activity and outcome parameters than cross-sectional studies. Furthermore, we had a low compliance of 31.2% in CRF, which was predominantly caused by the limitations of the Åstrand test protocol, such as weight exceeding the bike limit, physical complaints (e.g. joint injuries), health issues (e.g. severe hypertension) and use of beta blockers. Nevertheless, compliance rate was not different across PA subgroups (~30% of each subgroup), suggesting no selection bias and leaving a sufficient sample size (*n* = 1232) to determine the relation between change in physical activity and CRF.

## Conclusion

Bariatric patients who managed to achieve improvements in habitual physical activity up to two years post-surgery had better body composition, fitness and health-related quality of life at 2 years post-surgery, compared to patients who maintained or even reduced their PA levels. These findings underscore the importance of perioperative bariatric care programs to change lifestyle and achieve sustainable improvements in PA levels.

## Electronic supplementary material

ESM 1 (JPG 1.27 mb)

ESM 2 (JPG 2.79 mb)
