# Supplementary figures and images for: Changes in Physical Activity in Relation to Body Composition, Fitness and Quality of Life after Primary Bariatric Surgery: a Two-Year Follow-Up Study
Source: Obes Surg. 2020 Oct 3;31(3):1120–8. doi: 10.1007/s11695-020-05009-x (PMC8249277; doi:10.1007/s11695-020-05009-x)

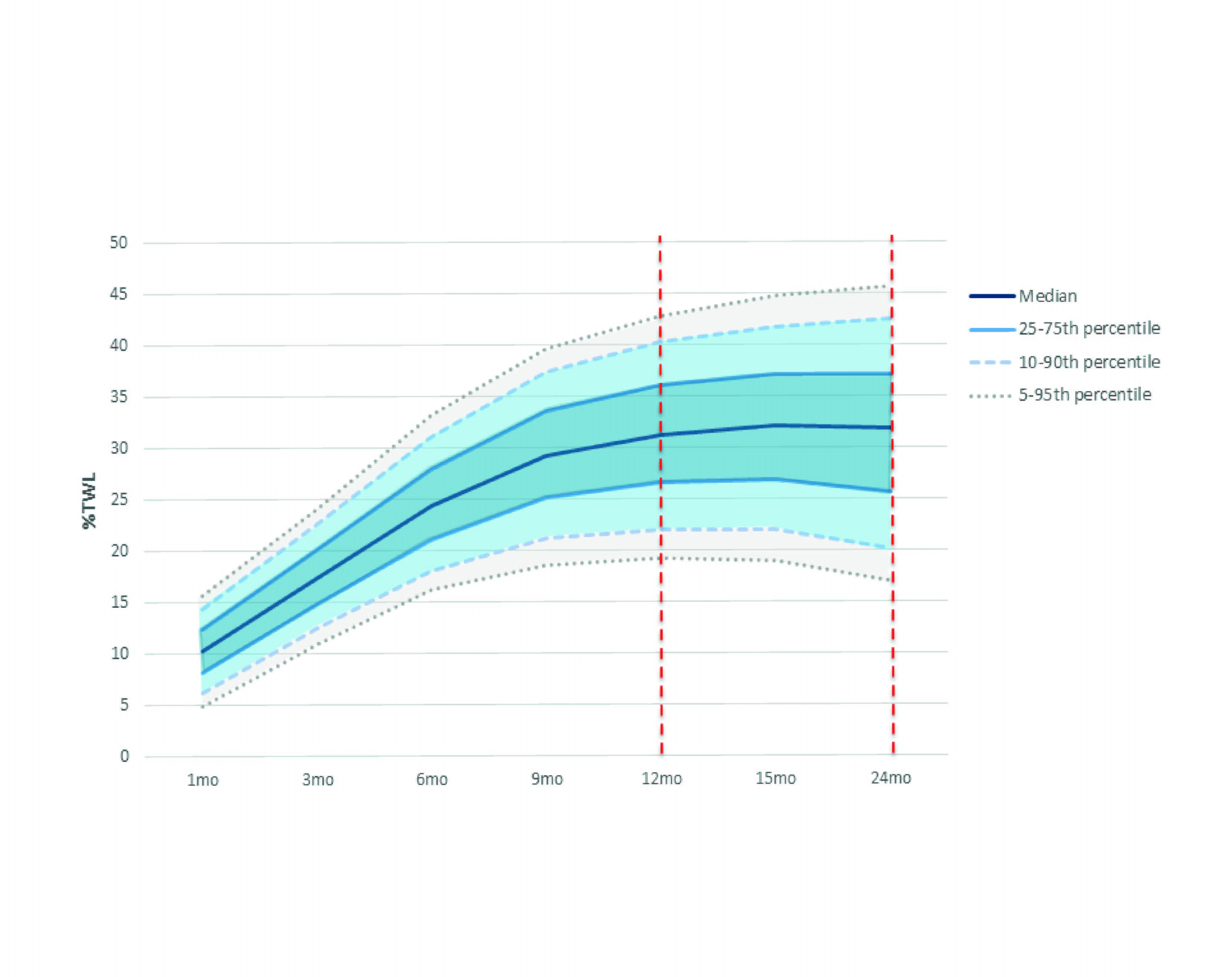

Supplement: Supplementary file 1 — (JPG 1.27 mb) [file 11695_2020_5009_Fig1_ESM.png]

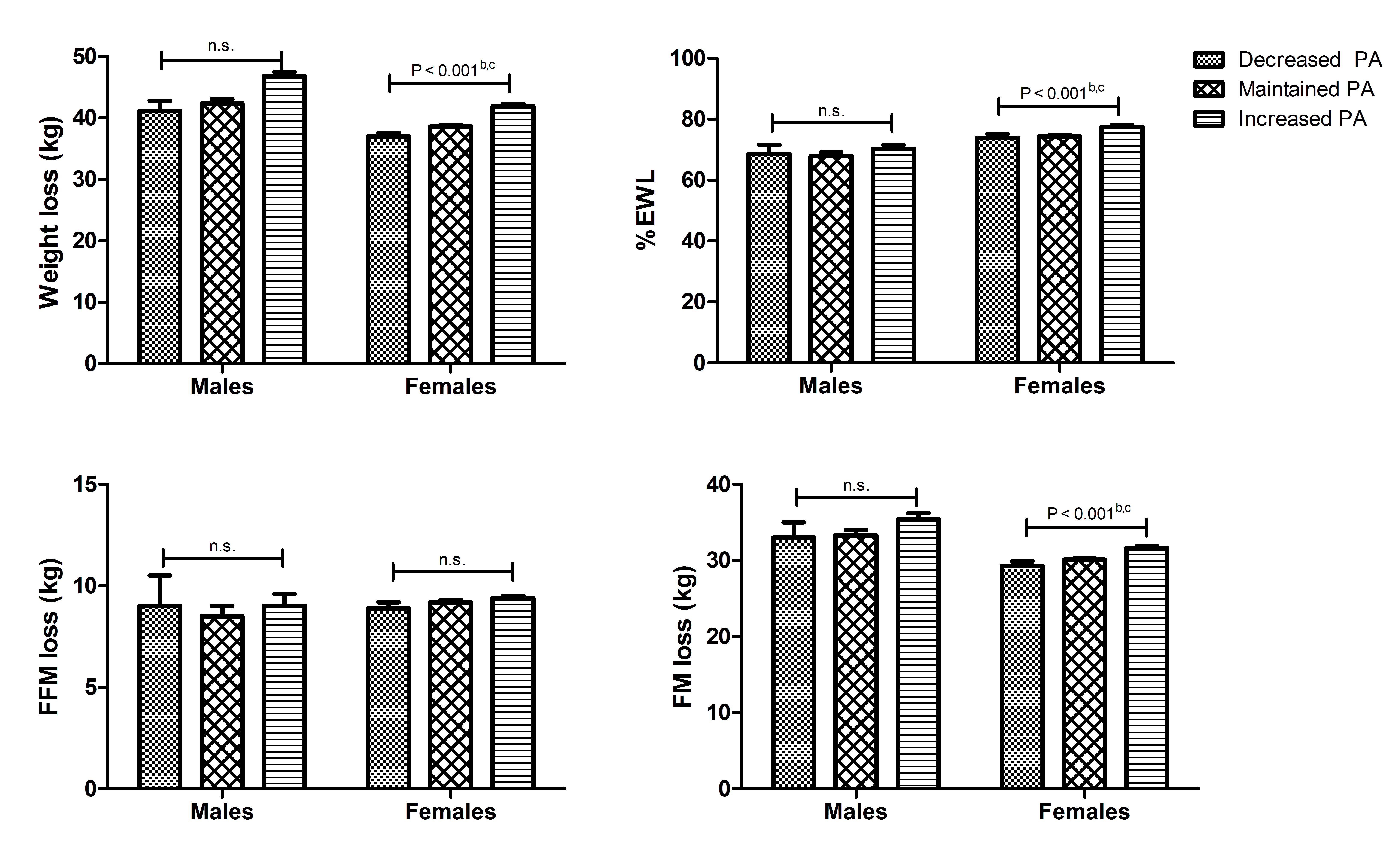

Supplement: Supplementary file 2 — (JPG 2.79 mb) [file 11695_2020_5009_Fig2_ESM.png]
